# Gene regulatory behaviors of mutant CRX proteins demonstrated with late-stage human retinal organoids

**DOI:** 10.1093/hmg/ddag107

**Published:** 2026-10-15

**Authors:** Nathaniel K Mullin, Andrew P Voigt, Deepak A Lamba, Kathleen R Chirco

**Affiliations:** Institute for Vision Research, Carver College of Medicine, University of Iowa, 200 Hawkins Dr, Iowa City, IA, 52242, United States; Department of Ophthalmology and Visual Sciences, Carver College of Medicine University of Iowa, 200 Hawkins Dr,Iowa City, IA, 52242, United States; Institute for Vision Research, Carver College of Medicine, University of Iowa, 200 Hawkins Dr, Iowa City, IA, 52242, United States; Department of Ophthalmology and Visual Sciences, Carver College of Medicine University of Iowa, 200 Hawkins Dr,Iowa City, IA, 52242, United States; Department of Ophthalmology, Feinberg School of Medicine, Northwestern Medicine, 303 E Chicago Ave, Chicago, IL, 60611, United States; Department of Ophthalmology, University of California San Francisco, 505 Parnassus Ave, San Francisco, CA, 94143, United States; Immunology and Regenerative Medicine, Genentech (Roche), 1 DNA Way, South San Francisco, CA, 94080, United States; Department of Ophthalmology, University of California San Francisco, 505 Parnassus Ave, San Francisco, CA, 94143, United States; Department of Ophthalmology, Casey Eye Institute, Oregon Health & Science University, 515 SW Campus Dr, Portland, OR, 97239, United States

**Keywords:** Leber congenital amaurosis, CRX, iPSC, retinal organoid, scRNA-seq, scATAC-seq

## Abstract

Variants in the transcription factor *CRX* cause a spectrum of retinal degenerative disorders including Leber congenital amaurosis 7 (LCA7). LCA7-associated variants exert a dominant effect in developing photoreceptor cells, leading to cellular dysfunction and poor to absent visual acuity in patients. Here, we dissect the allelic heterogeneity of LCA7 using multimodal single-cell gene expression and chromatin accessibility analysis of retinal organoids. We generated mature (240 day) retinal organoids from patient-derived iPSCs carrying missense (K88Q) and nonsense (T155fs) variants, as well as from CRISPR-corrected control lines. We show that these variants, while both causing LCA7, produce unique transcriptional changes in human photoreceptors. Furthermore, the K88Q variant causes aberrant chromatin accessibility at sites typically bound by non-photoreceptor retinal transcription factors, indicating a partial derangement of normal retinal cell fate programming. This work demonstrates the gene regulatory mechanisms of pathogenicity of two disease-causing *CRX* variants in human retinal cells.

## Introduction

Thousands of genetic variants in more than 250 different genes are known to cause inherited retinal disease [1]. These diseases affect the retina, a light-sensitive tissue responsible for converting light entering the eye to a neural signal that is transmitted to the brain via the optic nerve. Leber congenital amaurosis (LCA) is an inherited retinal dystrophy characterized by its congenital nature and poor to absent visual acuity. This condition is genetically heterogenous, with pathogenic variants in 14 genes accounting for approximately 70% of documented cases [2]. The genes that cause LCA encode proteins with a remarkably broad range of cellular functions and expression patterns across the diverse cell types of the retina including the light-sensing photoreceptors and the metabolically supportive retinal pigment epithelium (RPE) [2]. The phenotypic spectrum observed in LCA is broad, even between families carrying the same causal gene [2].

Many LCA genes are involved in photoreceptor cell development and function including *TULP1*, *CEP290*, *RPGRIP1*, *AIPL1*, *IMPDH1*, *RD3*, and *CRX*, the transcription factor that is the focus of this study. *CRX* encodes cone-rod homeobox (CRX, OMIM: 602225), a master transcriptional regulator responsible for inducing expression of genes essential to photoreceptor function and survival [3–5]. *CRX* expression is induced in OTX2-expressing photoreceptor precursors by day 80 of human fetal development [6] and maintained throughout life. While some causes of LCA have been shown to be amenable to gene replacement or oligonucleotide-based therapy in clinical trials [7–10], no treatment currently exists for *CRX*-associated disease.

Pathogenic variants in *CRX* cause a subclass of LCA known as LCA7 (OMIM: 613829), in addition to other retinal dystrophy phenotypes including cone-rod dystrophy [3, 11], macular dystrophy [12, 13], and autosomal dominant retinitis pigmentosa [3]. Most cases of *CRX*-associated retinal dystrophy are autosomal dominant, with rare recessive disease manifesting most often in an LCA phenotype [14]. There is significant allelic heterogeneity in *CRX*-associated disease, with 80 unique *CRX* variants currently reported as Pathogenic or Likely Pathogenic in ClinVar [4, 15]. Of these variants, most lead to either truncated proteins or amino acid substitutions [4]. Disease-causing *CRX* variants primarily disrupt the normal gene regulatory function of the transcription factor through alteration or ablation of either the DNA-binding homeodomain or transactivation domain [3]. While there is evidence for association between variant class and phenotype, both in terms of manifesting retinal dystrophy class and fundus appearance, the genotype–phenotype relationship for *CRX* remains incompletely understood [16].

Organoid-based modeling of human retinal development has become an increasingly valuable approach to study the early pathophysiology of inherited retinal disease [17–20]. Retinal organoids are known to recapitulate the molecular events of early retina specification as well as the anatomical organization of the neural retina and the maturation of cell type specific functions such as photoreceptor outer segment growth. Retinal organoids express *CRX* at the developmentally appropriate timepoint and within appropriate cell types [21]. The application of single-cell sequencing assays to human retinal organoids has further enabled description of the precise site and timing of abnormal gene expression downstream of retinal dystrophy variants [22]. Our group and others have previously applied such approaches to retinal organoids derived from *CRX* patients to identify early changes in photoreceptor gene expression [23, 24].

In this study, we explore the molecular basis of *CRX* genotype–phenotype association in LCA7. We examine gene regulatory targets of the intact protein as well as that of two *CRX* mutants to identify unique subsets of genes dysregulated in photoreceptors. Using single-cell studies of gene expression and chromatin accessibility, we delineate the genotype-specific changes to CRX function from a gene regulatory perspective. Through this approach, we define downstream regulatory effects that likely contribute to the congenital vision loss characteristic of LCA7 and identify nodes of potential therapeutic intervention in *CRX*-associated retinal dystrophy.

## Results

### Multimodal single-cell sequencing of late-stage LCA7 retinal organoids

We used previously described iPSC lines [24] derived from two LCA7 patients with severe phenotypes (Table 1) to investigate the gene regulatory effects of two *CRX* variants when expressed in developing photoreceptors, as well as the consequence of knockout of the mutant allele for one variant as an approach to phenotypic rescue (position of variants shown in Fig. 1A). One variant studied (K88Q) causes an amino acid substitution in the DNA-binding homeodomain while the other (T155fs) causes a frameshift and early truncation within the transactivation domain. A CRISPR-corrected control with two intact *CRX* alleles was generated from the K88Q line (referred to as ‘control’). Organoids from each line were differentiated to 240 days at which time nuclei were isolated and barcoded for joint single-cell RNA and ATAC sequencing (Fig. 1B). After computational filtering for high-quality nuclei, 10 485 total nuclei from four samples were included in the final dataset (Table 1). A median of 8160 unique transcripts and a median of 4322 genes were detected across all cells included in the analysis.

**Table 1:** Sample characteristics.

Sample	Sex	Age	Ocular History	Visual Acuity	*CRX Allele 1*	*CRX Allele 2*	Post-QC Cells
K88Q-isogenic control	F	3y	LCA	Light perception	WT (CRISPR-corrected)	WT	2680
T155fs	F	20y	LCA	Light perception	c.464_465insGGCA (p.T155ins4)	WT	2819
K88Q	F	3y	LCA	Light perception	c.262A>C (p.K88Q)	WT	2703
K88Qko	F	3y	LCA	Light perception	Exon3 KO	WT	2283

**Figure 1:**
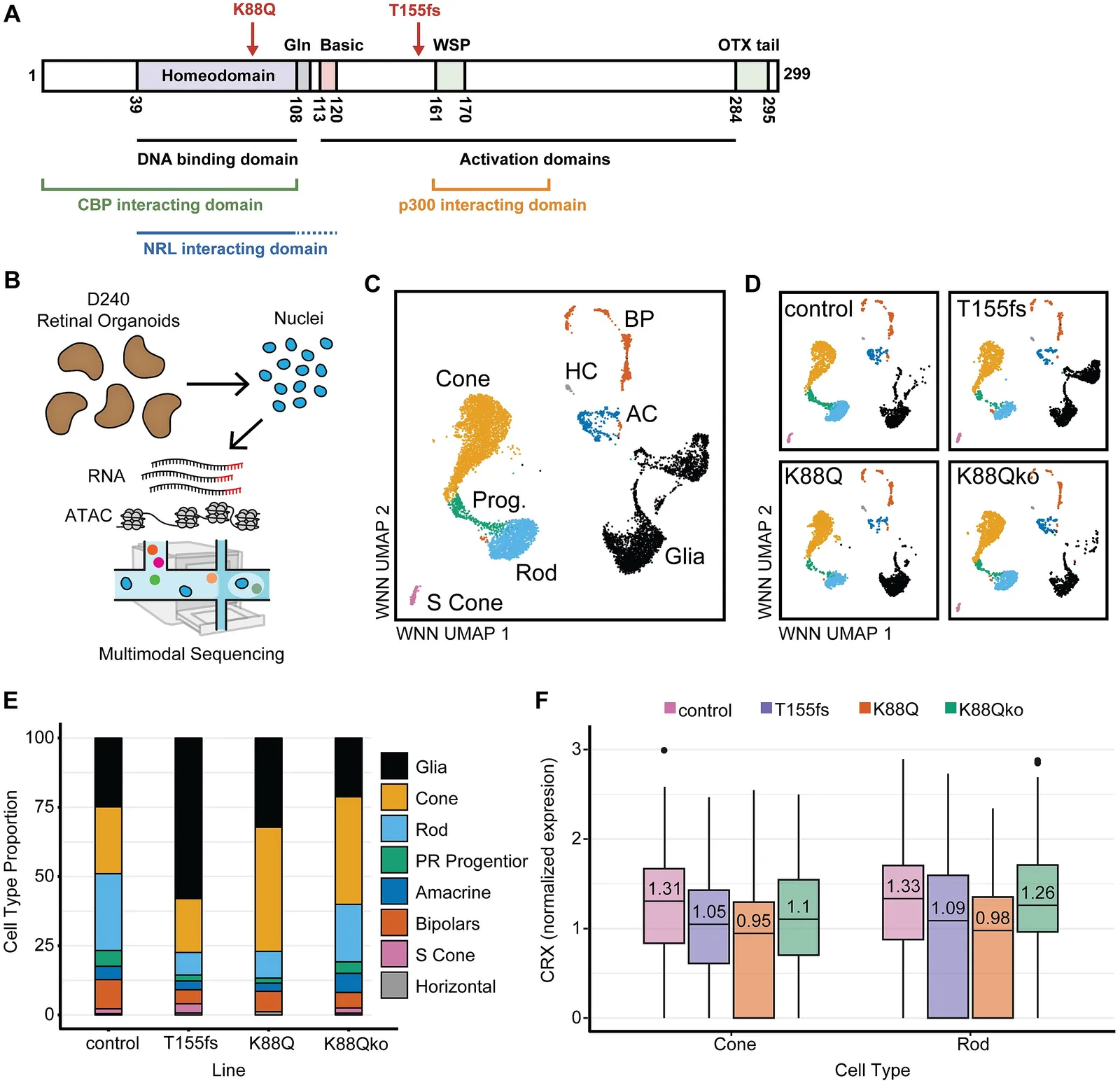
Composition of D240 retinal organoids measured by multiomic single-cell profiling. (A) Schematic showing the CRX protein map (1-299aa) with pathogenic variant locations indicated above in red. functional protein domains are highlighted and labeled. (B) Experimental schematic of the current study. (C) Cells are projected in two dimensions based on UMAP run on a weighted nearest neighbor (WNN) graph. WNN analysis was performed on RNA expression and chromatin accessibility (in the form of peak counts) in each cell. (D) Cells are shown projected in the same coordinates as C and are divided based on sample of origin. (E) the proportion of cell types recovered from each sample is shown. (F) The log-normalized RNA expression value of *CRX* within the rod and cone cluster for each line is shown. The median value is displayed. Box plot segments represent quartiles with outlier cells shown as dots. (See also figs S1 and S2).

### Cell type annotation of single cells from retinal organoids

Gene expression and chromatin accessibility data for each cell in the dataset were used in combination to drive clustering of similar cells across samples with Weighted Nearest Neighbor (WNN) analysis as previously described [25]. Cell types were annotated based on expression of canonical marker genes within these clusters (Fig. 1C, Fig. S2). Clusters that represented likely doublets expressing markers of multiple cell types were excluded from further analysis. As expected, our late-stage retinal organoids yielded primarily photoreceptor cells and glia, consistent with previous studies at comparable timepoints [21, 26–30] (Fig. 1C). The T155fs line produced a distinct population of glia not observed in other lines (Fig. 1D). To confirm that cell type clustering was not an artifact resulting from low-quality nuclei, post-filtering quality control metrics across annotated clusters and samples were visualized (Fig. S1). These metrics show no systemic deviation across samples, including within the glia cluster from the T155fs line.

### *CRX* patient lines and edited controls produce expected major inner and outer neural retina cell types following 240 days of culture

Using the cell type annotations shown in Fig. 1C, we next calculated the fraction of each cell type out of all cells captured from each sample, yielding a cell type proportion of analyzed cells (Fig. 1E). While all lines produced all annotated cell types (apart from K88Q which did not produce S cone photoreceptors), T155fs line yielded a higher proportion of glia. K88Q line yielded an increased proportion of cone photoreceptors at the apparent expense of rods, as compared to the isogenic control and K88Qko lines. No ganglion cells were observed in any lines, which is typical for 240-day-old organoids. All proportions are relative only to cells analyzed as absolute proportions and cell type counts are not attainable with scRNAseq.

### Expression of *CRX* is not significantly altered by *CRX* allelic status

We examined the expression level of *CRX* mRNA within photoreceptor cells from each line (Fig. 1F) to determine whether pathogenic *CRX* alleles diminished expression of mutant or wildtype CRX at the transcript level. While a range of expression in individual cells was found across samples (likely due, in part, to sub-optimal sampling inherent to the single-cell sequencing approach), the median expression value was not significantly altered between samples carrying wildtype, mutant, or knock-out alleles (Fig. 1E). For the purposes of this analysis, all *CRX* transcripts are mapped and counted towards the common *CRX* feature, meaning that transcripts originating from the K88Qko allele were included in this analysis.

### *CRX* T155fs and K88Q produce distinct changes in photoreceptor gene expression

We next focused on the gene expression profile of rod and cone photoreceptors due to CRX’s known function as an essential photoreceptor transcription factor. First, we assessed how *CRX* allelic status alters photoreceptor gene expression. Differential gene expression analysis was performed between the isogenic control line versus the T155fs, K88Q, and K88Qko lines across both the rod and cone clusters (Fig. 2A–C, and  G–I). For this analysis, genes with an absolute log_2_(fold change) greater than 1, adjusted p-value < 0.05, and detected expression in at least 10% of cells in either condition were considered as differentially expressed genes (DEGs). Within the rod cluster, T155fs, K88Q, and K88Qko exhibited 974, 1100, and 278 DEGs respectively (Fig. 2A–C). Within the cone cluster, T155fs, K88Q, and K88Qko exhibited 1082, 1215, and 604 DEGs respectively (Fig. 2E–G). The number of differentially expressed genes between each sample and control line, as well as the intersection of these sets was calculated (Fig. 2D, E, J, and  K).

**Figure 2:**
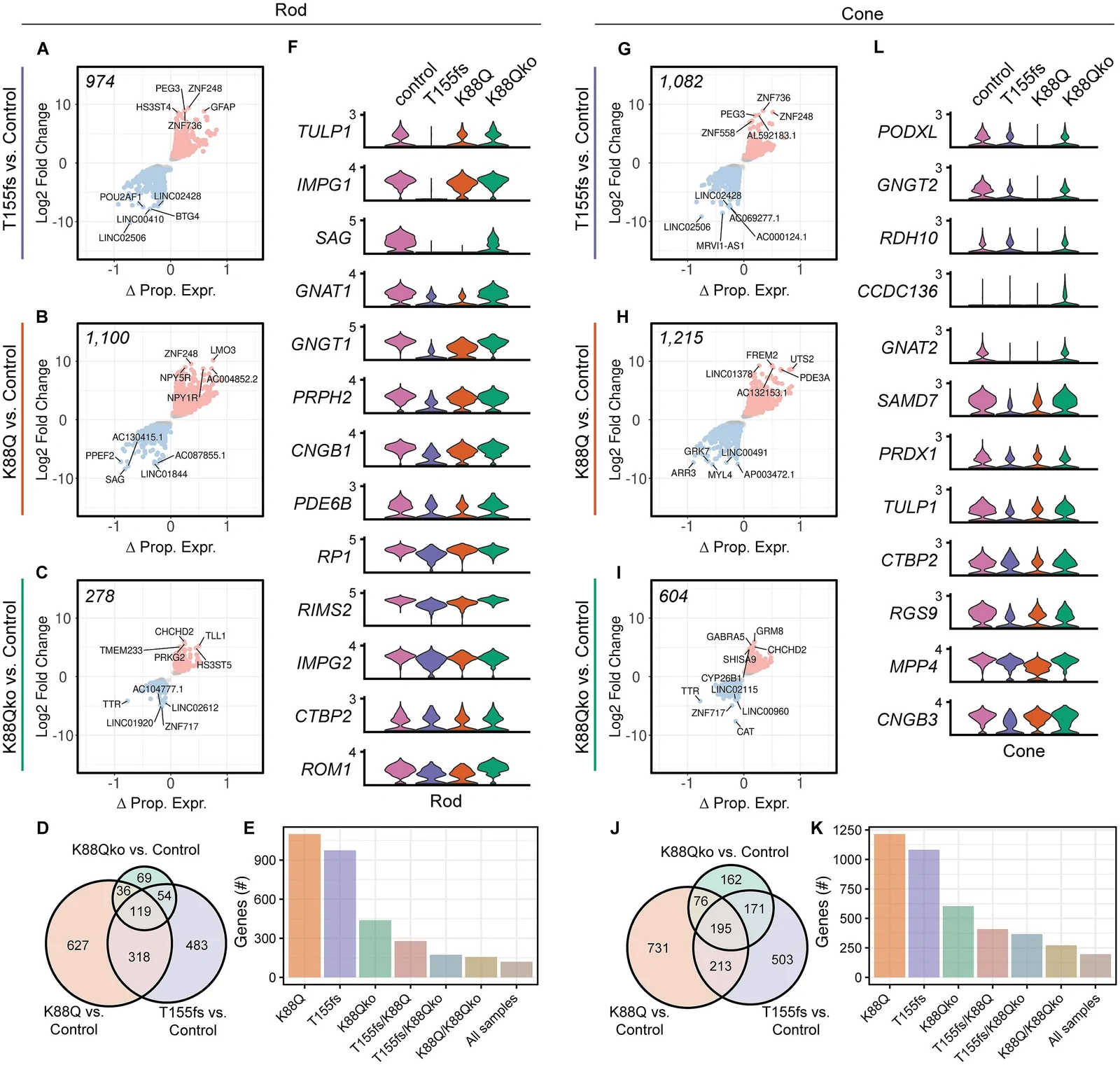
CRX functionality alters expression of specific photoreceptor genes in an allele-specific manner. (A–C) Differentially expressed genes within the rod cluster between T155fs, K88Q, K88Qko and the isogenic control are shown. Genes are plotted by log2(fold change) (y-axis) and the difference in proportion of cells expressing each gene between conditions (x-axis). Genes with an absolute log2(fold change) > 1 are colored in red or blue. The top 5 genes (based on fold change) in either direction are labeled. (D) The number of differentially expressed genes within the rod cluster between each test line and control and common between test lines is shown. (E) The number of differentially expressed genes within each set from D is shown. Genes may be counted in multiple columns if present in multiple sets. (F) Normalized gene expression of cells within the rod cluster is shown for each line. Genes shown are known markers of rod photoreceptor maturation. (G–I) Differentially expressed genes within the cone cluster between T155fs, K88Q and K88Qko lines and control are shown as in A–C. J As in D, a Venn diagram shows the number of differentially expressed genes unique and common to the three lines within the cone cluster. (K) The number of differentially expressed genes in each set is shown. (L) Normalized gene expression of cone photoreceptors is shown for each line. Genes shown are known markers of cone photoreceptor maturation. Significantly differentially expressed genes (i.e. those represented in D, E, J, and K) were defined as those with absolute log2(fold change) > 1 and adjusted *P*-value < 0.05 based on the Wilcoxon rank sum test with Bonferroni correction using all genes. Only genes expressed in at least 10% of cells in either comparison group were considered. (See also figure S3).

### Knockout of *CRX* K88Q partially restores normal photoreceptor gene expression

In both photoreceptor clusters, the K88Qko line was more transcriptionally similar to the isogenic control line than the unedited K88Q line (Fig. 2D, E, J, and  K), implying that K88Q DEGs were mostly due to a gain of abnormal function due to the CRX K88Q variant. However, a minority of genes remain differentially expressed following knockout, suggesting a set of genes sensitive to CRX haploinsufficiency. The majority of DEGs found in the K88Qko line were shared with one or both mutant lines (Fig. 2D and J).

### *CRX* variants impair expression of photoreceptor maturity marker genes in an allele-specific manner

CRX is known to regulate expression of essential photoreceptor genes during retinal development. Using the rod and cone populations, we next examined the expression of genes involved in photoreceptor maturation and function across the four lines. Within the rod cluster (Fig. 2D), two genes that were well expressed in the K88Q and control lines were poorly expressed in the T155fs line (*TULP1* and *IMPG1*). Two genes were poorly expressed in both mutant lines compared to controls (*SAG* and *GNAT1*). Within the cone cluster (Fig. 2H), *PODXL*, *GNGT2*, and *RDH10* were all expressed at a lower level in K88Q than the other mutant or control lines. Both T155fs and K88Q exhibited lower *GNAT2* and *SAMD7* expression compared to control. Most curated photoreceptor maturity markers of both rod and cone maturation were well expressed in mutant and control lines.

### CRX dysfunction alters photoreceptor chromatin accessibility

Regions of chromatin accessibility (‘*peaks*’) were defined by overlapped ATAC reads above a background level as previously described [31]. All cells of each cell type were pooled across lines for this analysis to reduce calling of genotype-specific peaks which would be difficult to distinguish from technical artifact. Accessibility at these peaks was then compared between samples across each cell type cluster (Fig. 3A and B). Aside from the large number of differentially accessible regions in the glia from the T155fs line (Fig. 3B), most of the variation in chromatin accessibility was restricted to rod and cone clusters (Fig. 3A). The K88Qko line displayed reduced deviation of accessibility compared to the isogenic control line in both clusters, highlighting the dominant negative functionality of this variant and corroborating what was observed at the transcript-level.

**Figure 3:**
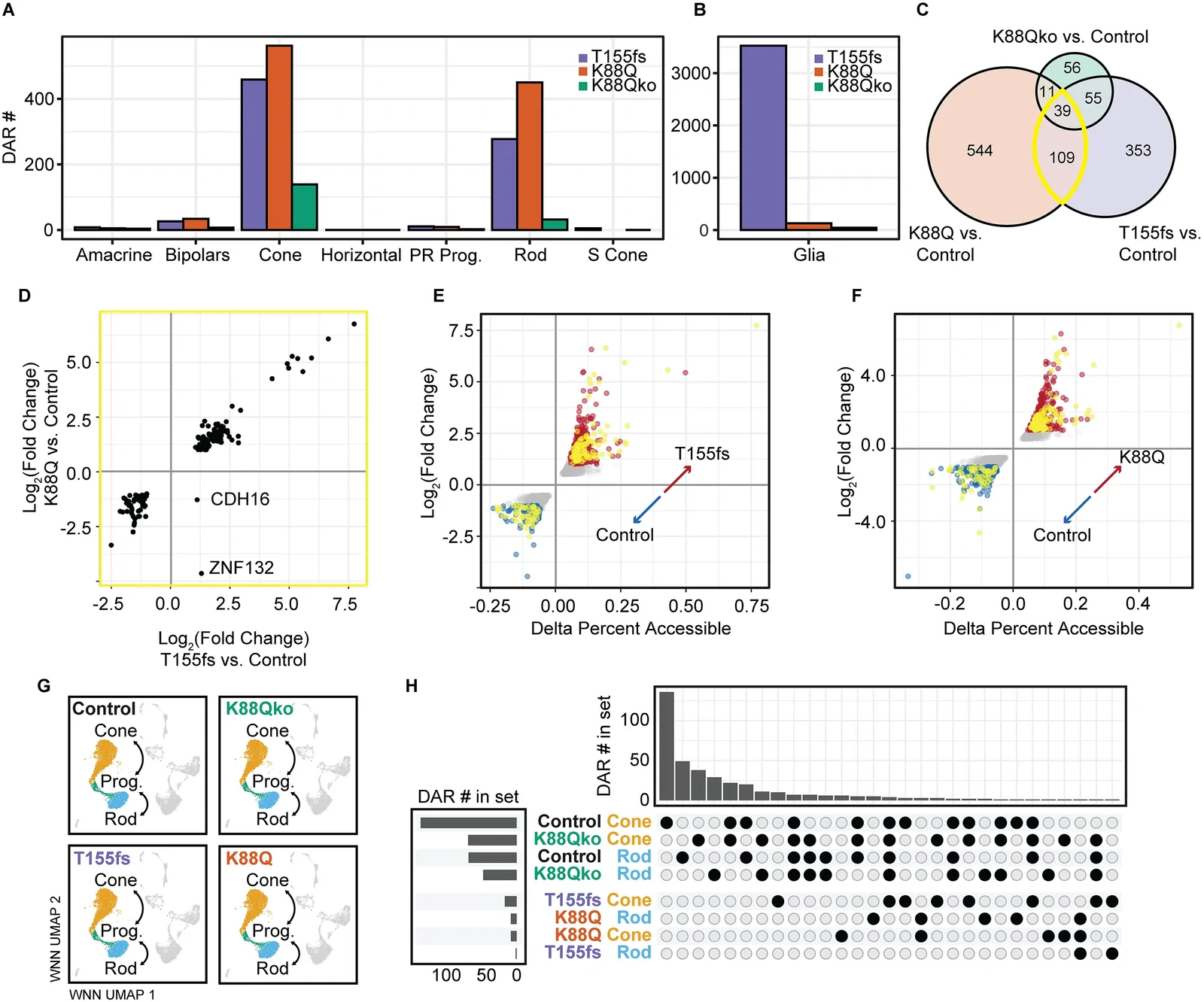
CRX functionality alters chromatin accessibility primarily in photoreceptor cells. (A) Differentially accessible regions (DAR) were identified between each sample and the control sample for each cell type identified. A high number of DARs were found in CRX mutant lines in the cone and rod clusters. (B) The glia of the T155fs line displayed a highly aberrant pattern of chromatin accessibility. (C) DARs shared between comparisons in A are shown in a Venn diagram. For this plot, cells of the cone and rod cluster were combined. DARs found in both the T155fs and K88Q lines are highlighted in yellow. (D) Average fold-change of accessibility of the DARs highlighted in yellow in C is plotted for both T155fs and K88Q compared to control. All but 2 DARs exhibit the same directionality in both mutant lines. (E and F) average fold change and the difference in proportion of cells with region accessibility are plotted for the T155fs and K88Q lines. Yellow dots indicate DARs that are common to both comparisons. Significantly differentially accessible regions were defined as those with absolute log2(FC) > 1 and adjusted *P*-value < 0.05 based on logistic regression model with likelihood ratio testing adjusted with Bonferroni correction using all peaks. Only genes regions identified in at least 1% of cells in either comparison group were considered. (G) Analytic schematic representing the comparisons shown in H. For each sample, rod and cone clusters were individually compared to that sample’s progenitor cluster (eight comparisons total). (H) DARs identified by the comparisons shown in G are represented in an UpSet plot. Horizontal bars represent the number of DARs identified in each sample’s photoreceptor clusters compared to progenitors of the same sample. Vertical bars represent the number of DARs that are unique or shared between comparison groups, indicated by shaded circles. Most DARs are found in the isogenic or K88Qko control lines.

We next compared differentially accessible regions (DARs) of chromatin from each sample to determine whether these were largely overlapping or allele specific. Within the photoreceptor clusters (i.e. rod and cone clusters), most variation in chromatin accessibility was restricted to a single sample. The T155fs and K88Q lines each respectively had 353 and 544 differentially accessible regions compared to the same control (Fig. 3C). A total of 148 regions of differential accessibility were shared between these lines (Fig. 3C, in yellow). All but 2 of these peaks exhibited the same directionality of differential expression in both T155fs and K88Q lines (Fig. 3D), implying a core set of responsive regions downstream of normal CRX function. DARs identified in each comparison are highlighted in Fig. 3E and F, with common DARs shown in yellow. Both *CRX* T155fs and K88Q variants lead to both increased and decreased accessibility at several sites across the genome compared to the control line.

To evaluate whether abnormal CRX function leads to a stalling of normal photoreceptor development, we compared chromatin accessibility between rod and cone clusters and the photoreceptor progenitor cluster within each line (Fig. 3G). We found far more DARs in the control and K88Qko comparisons than within the K88Q and T155fs (Fig. 3H), indicating that the chromatin accessibility landscape of photoreceptors with mutant CRX may fail to mature in a typical fashion. Knockout of the K88Q allele resulted in DARs largely overlapping with those observed in the isogenic control.

### The K88Q substitution in CRX causes increased accessibility of VSX2-family motifs

We next asked how the K88Q variant alters normal CRX target gene regulation at the transcript and chromatin level. This variant leads to an amino acid substitution from lysine to glutamine at relative position 50 within the DNA-binding homeodomain of CRX [32, 33]. Substitution at this position is known to discriminate CRX and OTX2 factors from RAX/VSX2/ARX family factors [34] and is important to the differentiation of photoreceptor and bipolar cell gene expression wherein Q50 homeodomain factors normally suppress expression of photoreceptor-specific genes [34]. The consensus motifs for CRX (Fig. 4B), VSX2 (Fig. 4B) differ based on this homeodomain difference.

**Figure 4:**
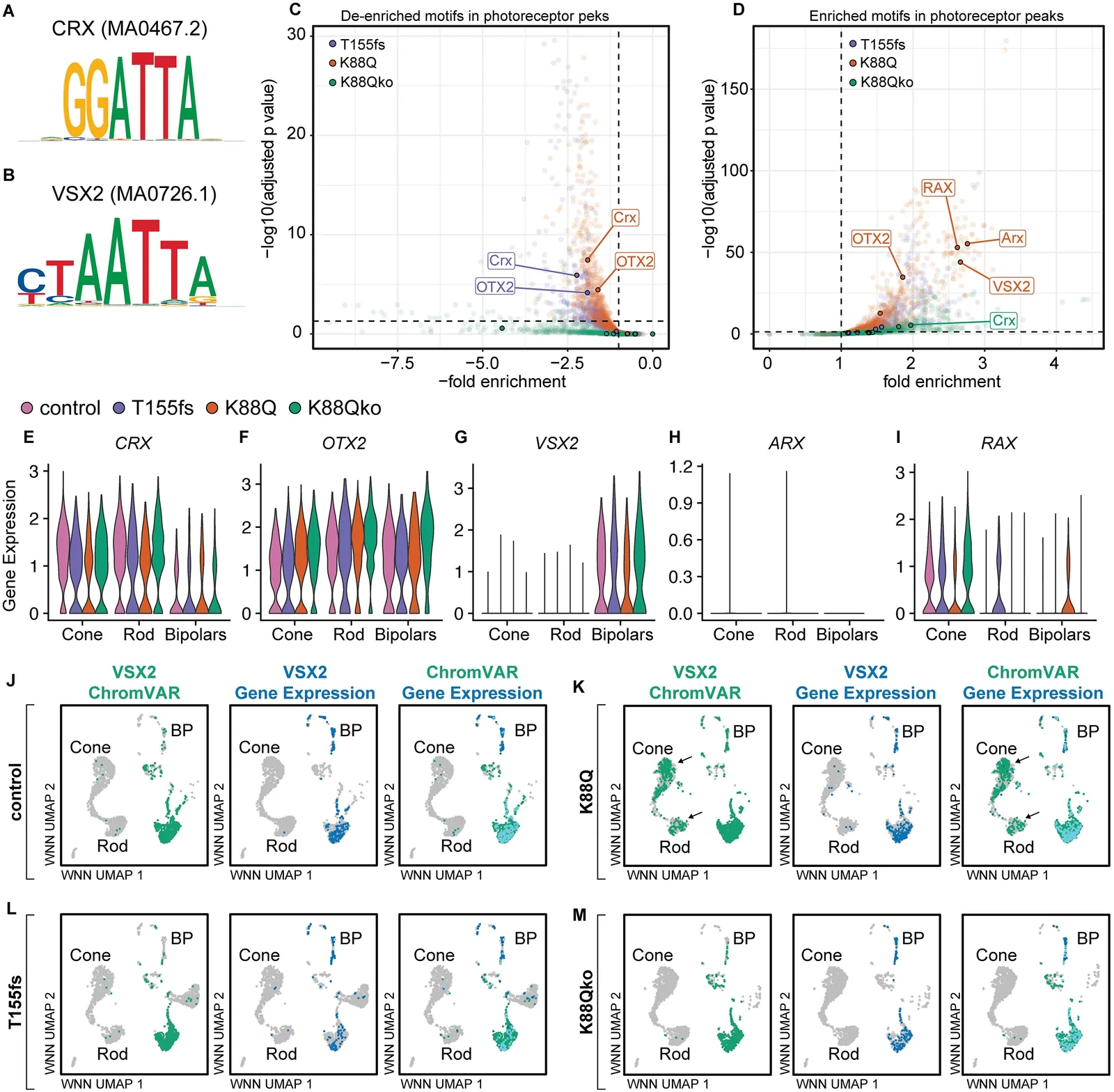
The *CRX* K88Q variant induces aberrant *VSX2*-family motif accessibility in photoreceptors. (A and B) Sequence logos for CRX and VSX2. (C and D) Enrichment of the motifs shown in A and B, as well as related factors, within photoreceptor peaks in each sample is shown. Enrichment is calculated as the percentage of differentially accessible ATAC peaks (between test photoreceptors and control photoreceptors) containing the motif of interest divided by the percentage of background peaks containing the same motif. Background peak sets are non-differentially accessible photoreceptor peaks matched for GC content. In C, both K88Q and T155fs lines display de-enrichment of CRX and OTX2 motifs in photoreceptor peaks. In D, the K88Q line shows enrichment of ARX/RAX/VSX2 motifs within photoreceptor peaks. (E–I) Normalized gene expression of the above transcription factor genes is shown in cone, rod, and bipolar cell populations from the four samples assayed. Minimal expression VSX2/ARX/RAX is detected in photoreceptor cells, consistent with normal developmental expression patterns. (J) UMAP projection of control cells is shown colored by VSX2 motif activity (green, left), *VSX2* gene expression (blue, center), and both modalities (right). In the control line, minimal VSX2 motif activity nor gene expression is detected in cone or rod clusters. (K) In the K88Q line, VSX2 ChomVAR motif activity is detected in both rod and cone photoreceptors (black arrows), but gene expression is not found in photoreceptor populations. (L and M) Both the T155fs and K88Qko lines display normal patterns of VSX2 motif activity and gene expression. (See also figure S3).

We used gene expression and chromatin accessibility data from photoreceptors carrying the CRX K88Q allele to ask how this disease-causing variant leads to malformation of photoreceptors. We first identified the set of DARs between each line and control within rod and cone clusters as described above (i.e. those in Fig. 3A). We next computed enriched and de-enriched transcription factor binding motifs within each set of peaks compared to a set of background peaks matched for GC-content (Fig. 4C and D). In both mutant lines (T155fs and K88Q) we found that *CRX* and *OTX2* motifs were de-enriched, while the K88Qko line did not display de-enrichment of these motifs within its differentially accessible peaks (Fig. 4C). We examined motifs that were enriched in each differentially accessible peak set. *RAX*/*ARX*/*VSX2* motifs were significantly enriched within the K88Q line but not enriched in the other tested lines (Fig. 4D).

### *CRX* variant presence does not alter *CRX* or *OTX2* expression in developing photoreceptors

To confirm that our organoids exhibited the expected developmental pattern of *CRX*, *OTX2*, and *VSX2* gene expression, we examined expression of these genes, as well as *ARX* and *RAX* within cone, rod, and bipolar clusters for all 4 lines (Fig. 4E–I, Fig. S4). *OTX2* was expressed in photoreceptors and bipolars in all lines, while *CRX* was primarily restricted to photoreceptors (Fig. 4E and F). Notably, *VSX2* was expressed only in bipolars in all lines including K88Q (Fig. 4G). As *VSX2* transcript was not detected in photoreceptor cells, the aberrant enrichment of *VSX2* motifs observed in this cluster (Fig. 4D) is likely not due to the presence of VSX2 itself but instead may be due to inappropriate binding of mutant CRX to this motif.

### VSX2 motif activity and gene expression in single cells

We next applied a per-cell approached to investigate the co-occurrence of transcription factor gene expression and motif accessibility leveraging our multiomic dataset. Motif activity scores were generated for each cell in the dataset using ChromVAR [35]. The per-cell motif activity score for VSX2 as well as the magnitude of *VSX2* RNA expression are shown (Fig. 4J–M). Normally, neither *VSX2* expression nor motif activity are detectable in photoreceptor cells (Fig. 4J). In the K88Q line, *VSX2* motif activity is seen in both rod and cone clusters (Fig. 4K). Notably, these clusters do not demonstrate expression of *VSX2* RNA. No *VSX2* motif activity is seen in T155fs photoreceptors (Fig. 4L), and knockout of K88Q also abolishes presumed aberrant *VSX2* motif activity (Fig. 4M). CRX motif chromVAR activity was decreased in cones in mutant lines and decreased in rods in the T155fs line (Fig. S3). K88Q rods and K88Qko rods and cones exhibited comparable activity levels to the control line. Bipolar cells from all samples exhibited lower CRX activity compared to photoreceptors.

## Discussion

In this study, we aimed to define the gene regulatory changes downstream of two pathogenic *CRX* variants that cause autosomal dominant retinal dystrophy through different molecular mechanisms. We further explored the effects of a CRISPR knock-out approach to rescue dominant-negative variants [24], asking whether late-stage organoids with a knocked-out allele would be transcriptionally normal. While the gene regulatory changes induced by different CRX variants have been studied previously in animal models [32, 36] and using *in vitro* assays [37], we utilize retinal organoids differentiated from LCA7 patients to model disease mechanisms in human retinal cells with genetic backgrounds known to be permissive of CRX disease.

We show that both K88Q and T155fs variants lead to abnormal differentiation of retinal organoids in terms of cell type proportion produced, in agreement with ample previous evidence of the essential role CRX plays in photoreceptor development. We found that K88Q and T155fs led to derangement of largely unique gene sets as well as a set of common regulatory targets. This implies that in these pathogenic CRX variants, a common CRX function may be lost while variant-specific regulatory targets may also be created. It is known that pathogenic CRX variants cause distinct molecular phenotypes and do not necessarily cause disease through haploinsufficiency [38].

Chromatin accessibility analysis of photoreceptors carrying each variant further supports the concept of unique regulatory targets for K88Q and T155fs. Chromatin accessibility changes were largely restricted to cone and rod photoreceptor cells, though changes were also observed in the Glia population of T155fs organoids. Of the genomic regions that were differentially accessible in both mutant lines, we found that directionality was shared for most regions, indicating a common mechanism of regulatory disruption. As in the gene expression analysis, a large majority of differentially accessible regions were not shared between K88Q and T155fs, again supporting unique regulatory targets for these two variants.

Finally, we used two computational approaches to analyze the sequences of differentially accessible chromatin regions for enrichment of transcription factor binding motifs. Both K88Q and T155fs photoreceptors predictably showed an enrichment for CRX and related motifs in those peaks that were inaccessible compared to control lines, indicating a dominant negative loss of function. K88Q strikingly showed enrichment for VSX2-family motif accessible regions compared to control. This change was not observed in the T155fs line nor the K88Qko line, showing specificity of this effect to the K88Q variant. *CRX* K88N has previously been shown in mouse models to increase CRX binding to Q50 sites canonically bound by VSX2-family factors [32, 33]. The K88N substitution in human CRX is known to increase binding to RAX/VSX2/ARX family motifs [4]. While OTX2 is expressed in the retinal progenitors that give rise to both photoreceptor and bipolar cells, CRX expression is normally restricted to photoreceptors while VSX2 is restricted to bipolar cells and their progenitors [39]. We now show that disruption to the same residue within the homeodomain of human CRX causes inappropriate accessibility to VSX2 motifs in mature photoreceptors, despite lack of endogenous *VSX2* expression.

This study is limited by several factors common to organoid-based modeling of rare disease. Only single individuals with each genotype and single timepoints of organoid differentiation were included. While inclusion of the isogenic control line provides an ideal control for the K88Q and K88Qko lines, the effects of genetic background cannot be ruled out as a major confounding factor when comparing the T155fs line to the K88Q and controls, and further analysis of an isogenic control for the T155fs line would be required to draw biological conclusions from such comparisons.

Variability between differentiation batches is known to be present in retinal organoids, though to a lesser degree than intra-donor variability [40], Our study did not include samples from multiple rounds of differentiation. Further, while we captured sufficient numbers of nuclei to sample the major well-defined cell types of the neural retina, we cannot exclude genotype-related changes to rarer cell types not sufficiently sampled. For example, the differential observation of s-cone photoreceptors between lines here cannot be attributed to genotype without further representative sampling, as s-cones are known to be the rarest photoreceptor type both *in vivo* and in organoids. Additionally, the relation of the glial cell population observed in the T155fs line cannot be attributed to the *CRX* variant without an isogenic control. Instead, this population may represent a skewed differentiation potential intrinsic to the iPSC line. Since *CRX* is not typically expressed in retinal glia, this is more likely. Future experiments including unrelated individuals carrying the same variant will be useful to more fully define the set of genes subject to misregulation by each *CRX* variant.

While our study explored chromatin architecture through ATAC, direct measurement of CRX binding to defined regulatory elements using ChIP-seq, CUT&RUN, or similar approaches would provide more definitive evidence of CRX K88Q binding to VSX2-family motifs. Additionally, future studies will be required to investigate the impact of these CRX variants on binding to co-activator proteins (e.g. CBP and p300), which could help explain the observed changes to chromatin accessibility and transcriptional activation at some loci. Finally, while CRX is not known to be involved in retinal Glia cell development, we observed a large cluster of presumptive Glia in the T155fs sample. While this cluster was not the focus of the current experiment, further investigation of these cells is warranted.

## Materials and methods

### Retinal organoid differentiation

iPSC lines were cultured as previously described [24] and cryopreserved in mTeSR Plus medium (Stemcell Technologies, 5825) with 10% DMSO. For each line (control, T155fs, K88Q, and K88Qko), retinal organoid differentiation was initiated using cells at passage 56, 35, 30, and 65 respectively. iPSC quality control was confirmed as previously described [24]. Organoids were generated using a previously published protocol [24]. At day 240 (D240) of differentiation, organoids were snap frozen (without culture medium) and stored at −80°C until processing for single nucleus sequencing, as described below.

### Nucleus isolation from organoids

Nuclei were isolated from snap-frozen organoids using a protocol based on 10× Genomics’ demonstrated protocol CG000366. In brief, chilled 0.1× Lysis Buffer (Tris–HCl (10 mM), NaCl (10 mM), MgCl2 (3 mM), Tween-20 (0.01%), Nonidet P40 Substitute (0.01%), Digitonin (0.001%), BSA (1%), DTT (1 mM), RNase inhibitor (1 U/μL)) was added to each sample. Samples were homogenized using a sterile pestle. The resulting mixture was incubated on ice for 5 minutes, then mixed 10 times with a P1000 pipette tip. The mixture was incubated on ice for 8.5 minutes. 500 μL chilled Wash Buffer (Tris–HCl (10 mM), NaCl (10 mM), MgCl2 (3 mM), BSA (1%), Tween-20 (0.1%), DTT (1 mM), RNase inhibitor (1 U/μL)) was then added and mixed with a P1000 pipette five times. The nuclei were pelleted at 500× g for 5 minutes at 4°C. Washing was repeated in this manner for a total of three times. Following the final centrifugation, wash buffer was removed, and nuclei were resuspended in Diluted Nuclei Buffer (10× Genomics Nuclei Buffer (1× from 20× stock)), DTT (1 mM), RNase inhibitor (1 U/μL). Nuclei were counted manually using a hemocytometer and processed immediately using the Chromium Next GEM Single Cell Multiome ATAC + Gene Expression v1 system (10× Genomics).

### Library preparation, sequencing, and data processing

Final libraries were generated according to Chromium Next GEM Single Cell Multiome ATAC + Gene Expression User Guide CG000338 (10× Genomics, Rev E) without deviation. The libraries were quantified using the Qubit dsDNA HS Assay Kit and diluted to 3 ng/μL. Library quality was checked using the Bioanalyzer or Tapestation (Agilent). Raw sequencing reads were mapped to the GRCh38 reference genome provided by 10× Genomics (GENCODE Release 32 (GRCh38.p13)) without modification using CellRanger ARC v2.0.0 (10× Genomics) using default settings.

### Sample integration and clustering

Gene expression and chromatin accessibility datasets from the four samples were filtered for high quality nuclei and integrated count matrices were generated. Gene expression data were integrated using canonical correlation analysis using Seurat [41] and chromatin accessibility data were integrated using latent semantic indexing using Signac [42]. Dimensionality reduction was performed on both integrated datasets separately. Only nuclei passing quality control thresholds in both gene expression and ATAC modalities were included in the final, multimodal dataset.

RNA and ATAC data for each nucleus was used to drive clustering using weighted nearest neighbor analysis (Signac). Dimensions 1:30 of the gene expression PCA and dimensions 2:50 of the chromatin accessibility LSI analysis were taken as WNN input. The resulting ‘weighted’ shared nearest neighbor graph was used to identify clusters of nuclei using the smart local moving (SLM) algorithm [41] with a resolution of 0.5.

### Cluster cell type annotation

Annotation of the above clusters was determined manually based on marker gene expression and comparison with previously published single-cell RNA sequencing data from developing human fetal retina [21]. The identity of three clusters was not determined. DoubletFinder [43] was used to identify one of these clusters as likely doublets originating from photoreceptors and glia cells. This and the other two unidentified clusters were dropped from further analysis.

### ATAC peak calling, differential chromatin accessibility analysis, and motif enrichment

ATAC peaks to be used in motif enrichment analysis were first called using MACS2 [31, 44] as implemented by the CallPeaks function in Signac. Cells were grouped by cell type for peak calling. Cells from all lines were pooled for peak calling. To identify transcription factor binding motif enrichment, the FindMarkers function was used with the following parameters: only.pos = FALSE, test.use = LR, latent.vars = nCount_macs2_peaks. Differentially accessible regions were filtered for an adjusted p-value less than 0.05 and absolute Log2(Fold change) > 1. Enriched motifs were identified in differentially accessible regions using the FindMotifs function, with a set of 50 000 GC-matched peaks accessible in the same cell type used as background. Enrichment over background and significance of motifs were plotted, highlighting the motifs of interest. ChromVAR was used to generate per-cell motif activity scores as implemented by Signac [42].

### Differential gene expression analysis

The FindMarkers function (Seurat) identified differentially expressed genes between samples within each cluster tested. Genes with a log2(fold change) of at least 1 and an adjusted p-value below 0.05 were considered significantly differentially expressed.

### Trajectory construction with slingshot

Slingshot [45] was used to quantify differing maturity within the photoreceptor and photoreceptor progenitor clusters. The Slingshot function was run taking WNN UMAP-derived embeddings as ‘input.’ The starting (progenitor) and end (cone, rod) clusters were given, and the following parameters were used: *stretch = 0.1*, *thresh = 0.01*. Two trajectories were identified. Curves were drawn based on these trajectories from which pseudotime values were derived and given to each cell.

### Statistics

All statistical testing for differential gene expression and chromatin accessibility was implemented using the Seurat/Signac package in R [41, 42]. For gene expression analysis, significantly differentially expressed genes were defined as those with absolute log2(fold change) > 1 and adjusted *P*-value < 0.05 based on the Wilcoxon Rank Sum test with Bonferroni correction using all genes. Only genes with detectable expression in at least 10% of cells in either comparison group were considered. For ATAC-seq analysis, significantly differentially accessible regions were defined as those with absolute log2(fold change) > 1 and adjusted *P*-value < 0.05 based on logistic regression model with likelihood ratio testing adjusted with Bonferroni correction using all peaks. Only peaks identified in at least 1% of cells in either comparison group were considered. For all experiments, 4 organoids were used per sample. Organoids were pooled prior to library preparation resulting in one sequencing library per sample. For statistical tests, individual cells were used as replicates.

#### Resource availability

Requests for further information and resources should be directed to and will be fulfilled by the lead contact, Kathleen R. Chirco; (chirco@ohsu.edu).

- **Materials availability:** This study did not generate new unique reagents.
- **Data and code availability:** Single-cell RNA and ATAC sequencing data have been deposited to the NCBI Gene Expression Omnibus (GEO) as [GEO: GSE262958] and are publicly available as of the date of publication. All original code has been deposited to GitHub and is publicly available at https://github.com/nkmullin/crx_multiome as of the date of publication. Any additional information required to reanalyze the data reported in this paper is available from the lead contact upon request.

## Supplementary Material

supplement_pdf_ddag107
