# Supplementary material for: Gene regulatory behaviors of mutant CRX proteins demonstrated with late-stage human retinal organoids
Source: Hum Mol Genet. 2026 Oct 15;35(21):ddag107. doi: 10.1093/hmg/ddag107 (PMC13669955; doi:10.1093/hmg/ddag107)
Supplement: Supplementary file 1 [file supplement_pdf_ddag107.pdf]

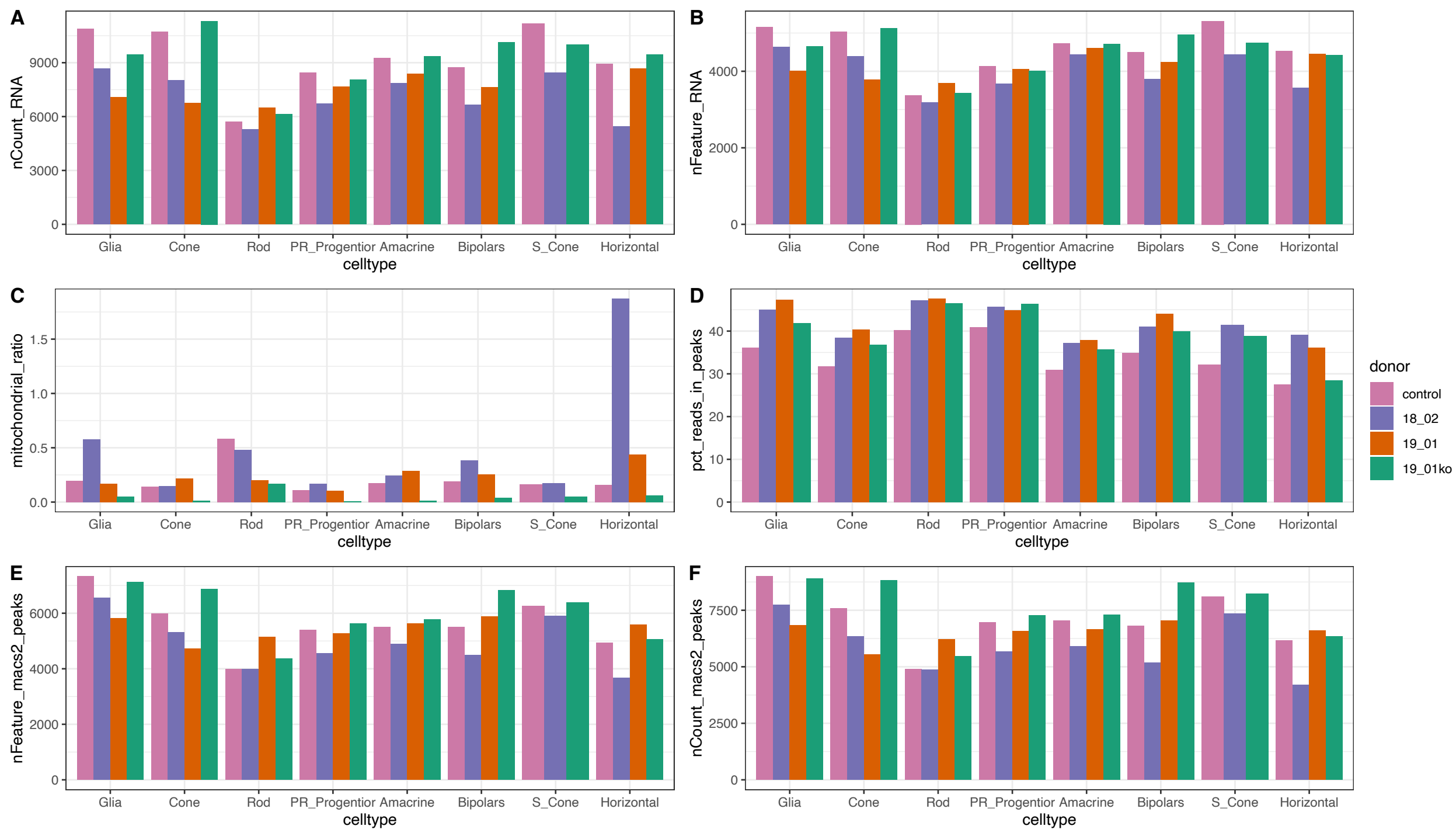

Figure S1. Median quality control metrics of analyzed cells. A) Median value of transcript unique molecular identifiers (nCount_RNA) for each cluster grouped by sample of origin is shown. Values represent only cells included in the final dataset used for analysis (i.e., after QC filters were applied, as described in Methods). **B)** Median values of the number of genes (features) detected per cell from the gene expression (RNA) arm of the experiment. **C)** Median value of the proportion of gene expression read mapping to the mitochondrial genome. **D)** Median value of the proportion of ATAC reads within called peaks (i.e. regions used for downstream analysis of chromatin accessibility). **E)** Median values of the number of peaks (features) detected per cell from the ATAC arm of the experiment. **F)** Median number of ATAC fragments mapped per cell.

A

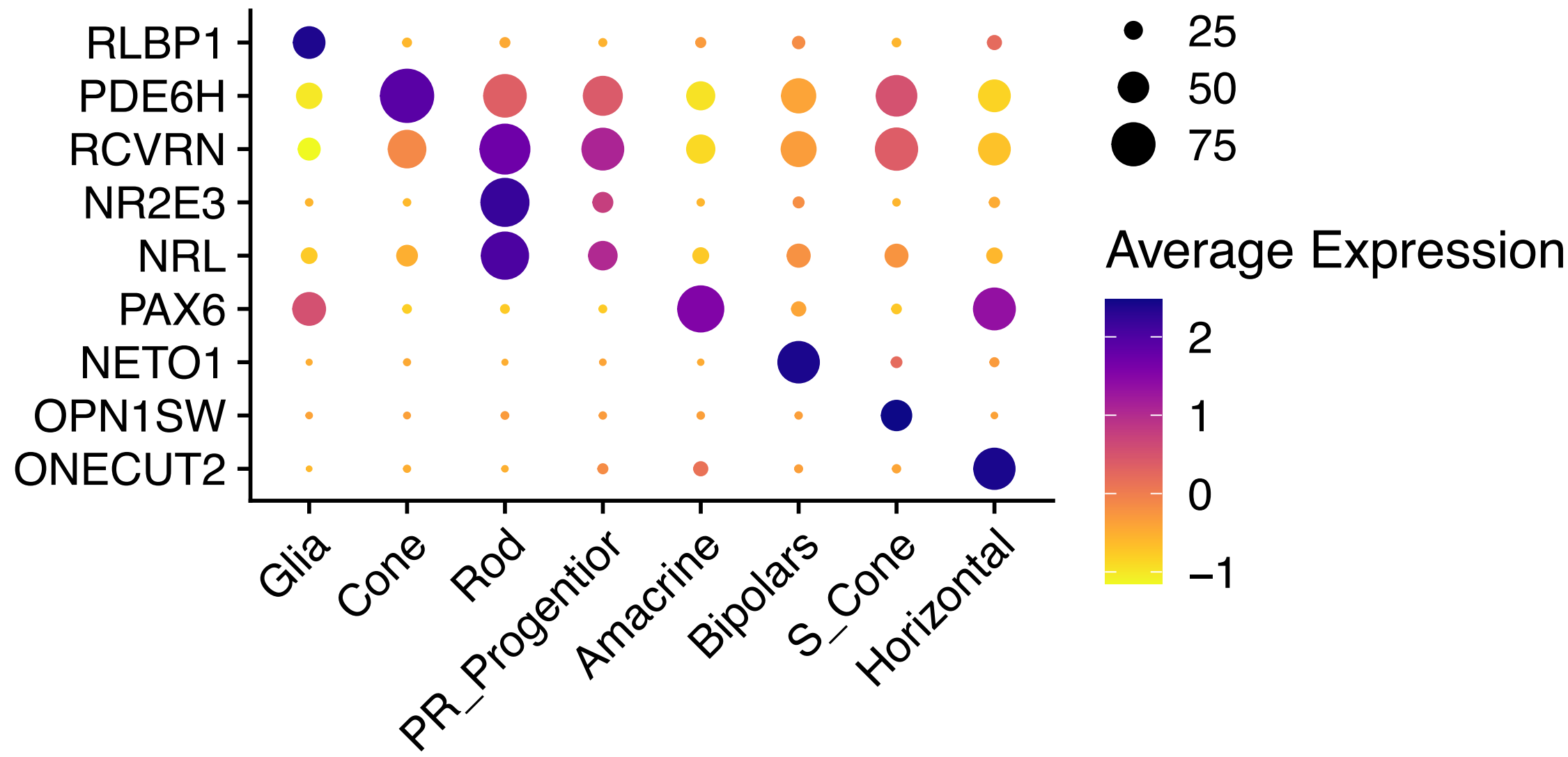

B

Gene Symbol	Gene Name	Canonical Retinal Cell Type Expression Pattern	Gene Function
RLBP1	Retinaldehyde-binding protein 1	Müller glia, Retinal pigment epithelium	Visual cycle
PDE6H	Phosphodiesterase 6H	Cone photoreceptor	cGMP phosphodiesterase, phototransduction
RCVRN	Recoverin	Rod and cone photoreceptor	Calcium sensor, phototransduction
NR2E3	Nuclear receptor subfamily 2 group E member 3	Rod photoreceptor	Transcription factor
NRL	Neural retina leucine zipper	Rod photoreceptor	Transcription factor
PAX6	Paired Box 6	Astrocyte, Müller glia, Amacrine, Horizontal	Transcription factor
NETO1	Neuropilin And Tolloid Like 1	Bipolar	Synaptic protein
OPN1SW	Opsin 1, Short Wave Sensitive	S-cone	G protein-coupled receptor, visual pigment
ONECUT2	One cut homeobox 2	Horizontal	Transcription factor

Figure S2. Dot plot of retinal cell type marker gene expression. A) Dot plot showing the percentage of cells (size) and magnitude of expression (color) of curated cell type-specific marker genes across annotated clusters shown in **Figure 1C**. All cells are included for each cluster regardless of *CRX* genotype. **B)** Table outlining the canonical localization and function of marker genes used in **A**.

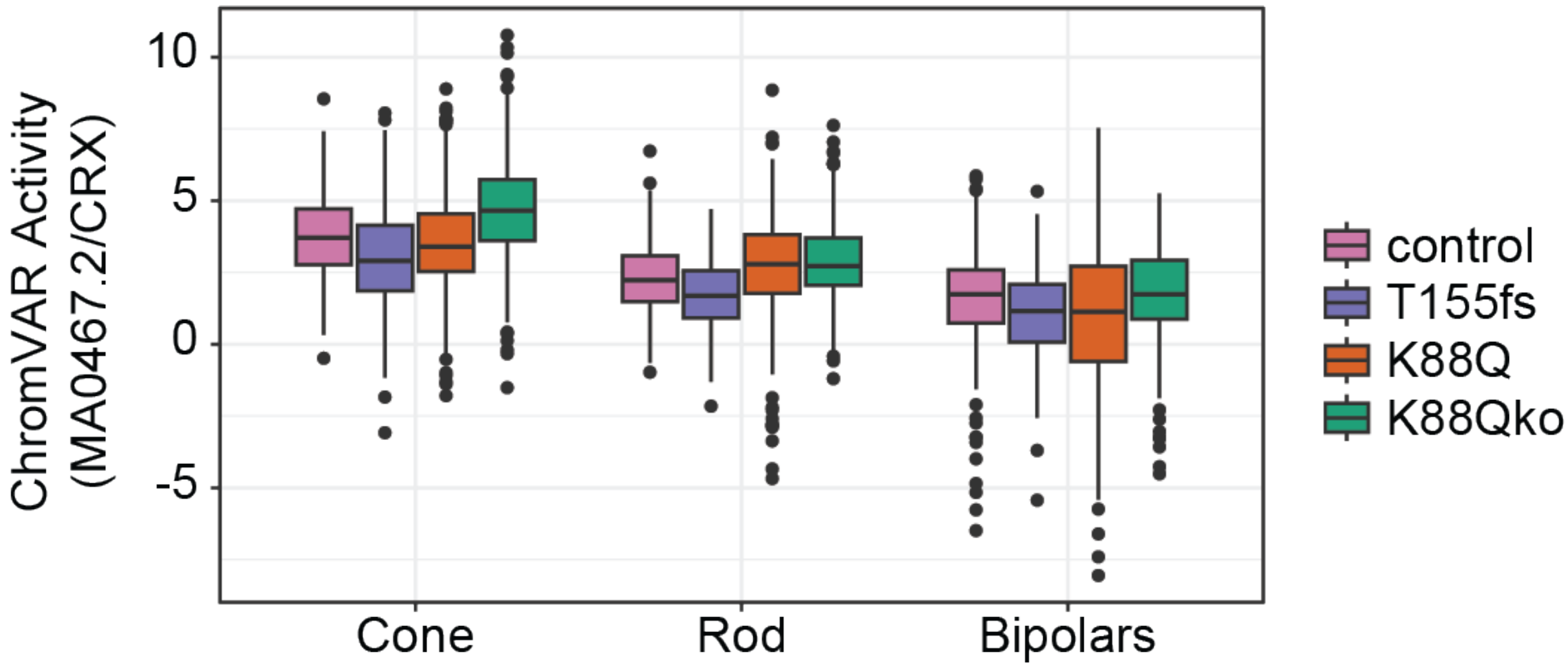

Figure S3. CRX motif chromVAR activity. Normalized chromVAR activity scores for the MA0467.2 CRX-binding motif are shown for cells within the photoreceptor and bipolar clusters for each sample.

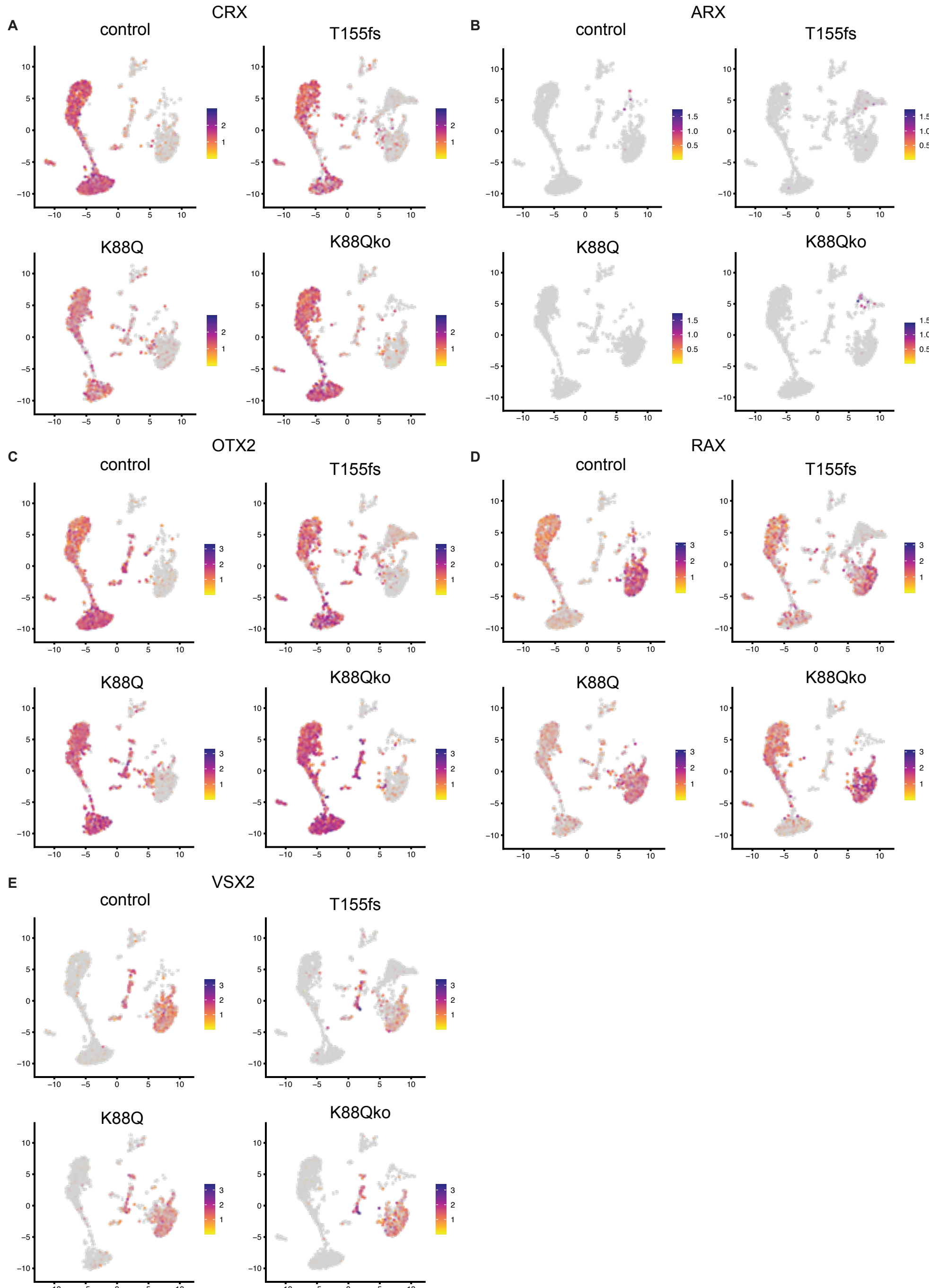

Figure S4. Per-cell transcription factor gene expression. A) Cells are shaded based on normalized expression level of CRX. B-E) As in A, cells are colored based on expression of ARX, OTX2, RAX, and VSX2 respectively. In all panels, cells are shown split by line/genotype of origin.
